# Subtotal stomach in esophageal reconstruction surgery achieves an anastomotic leakage rate of less than 1%

**DOI:** 10.1002/ags3.12336

**Published:** 2020-05-10

**Authors:** Kazuhiro Yoshida, Yoshihiro Tanaka, Takeharu Imai, Yuta Sato, Yuji Hatanaka, Tomonari Suetsugu, Naoki Okumura, Nobuhisa Matsuhashi, Takao Takahashi, Kazuya Yamaguchi

**Affiliations:** ^1^ Department of Surgical Oncology Graduate School of Medicine Gifu University Gifu Japan

**Keywords:** anastomotic leakage, esophageal surgery, quality of life, reconstruction, subtotal stomach

## Abstract

**Aim:**

The objective of this retrospective, single‐institution study was to assess the safety and feasibility of reconstruction using subtotal stomach (SS) with esophagectomy for patients with esophageal cancer (EC). Although several different gastric‐tube‐making and anastomotic methods have been reported, the incidence rate of anastomotic leakage with EC surgery is generally reported over 10%. Complications should be avoided, and patient quality of life (QOL) should be maintained postoperatively. We have used SS reconstruction and hand‐sutured cervical esophagus‐subtotal gastric anastomosis at the neck wound in EC surgery. Short‐ and long‐term outcomes in cases using SS are not well known.

**Methods:**

Between January 2008 and September 2019, 300 patients underwent esophagectomy for EC and reconstruction using SS. The primary endpoint was the rate of anastomotic leakage. Secondary endpoints were postoperative morbidities, QOL, and changes in patients’ body weight and skeletal muscle weight.

**Results:**

Anastomotic leakage was observed in two patients (0.67%), and pneumonia was observed in nine patients (3.0%). Fifteen patients (5.0%) had an anastomotic stenosis requiring a bougie. Nausea occurred in 11 patients (3.7%), and dumping syndrome occurred in seven patients (2.3%). Dysphagia and early feeling of abdominal fullness scores tended to be high after surgery but gradually decreased after 6 months. Good results were obtained for reflux feeling scores. Body weight changed with an average decrease of −2 ± 3.71 kg (*P* = .071) over 5 years.

**Conclusion:**

Reconstruction using SS resulted in an extremely low rate of anastomotic leakage and good QOL postoperatively in patients with EC.

## Graphical abstract

Reconstruction using subtotal stomach resulted in an extremely low rate of anastomotic leakage and good QOL postoperatively in patients with esophageal cancer.

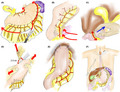

## 1 INTRODUCTION

The outcome of patients with esophageal cancer (EC) has generally improved due to advances in the diagnosis of early lesions using narrow‐band imaging^1^ and endoscopic treatment for such early cancer.^2^ Multidisciplinary treatment for advanced cases has also improved remarkably.^3^ However, postoperative complications of esophagectomy are reported to be 41.9%, and perioperative mortality is 3.4%, which is higher than that for other surgeries.^4^

Once anastomotic leakage develops after EC surgery, recovery may sometimes be difficult, which extends the hospital stay and physically exhausts the patients.^5^, ^6^ Advances in surgical equipment have increased the use of automatic suture devices to create gastrointestinal anastomoses. However, the incidence of anastomotic leakage in esophagectomy and reconstruction surgery is over 10%.^4^, ^7^ Avoidance of anastomotic leakage following EC surgery remains an important issue, with various methods reported in relation to their safety.^8^, ^9^ Also, because the anastomosis method affects postoperative quality of life (QOL), various devices to improve swallowing function and prevent reflux have been reported.^10^, ^11^, ^12^, ^13^, ^14^

The stomach is often used for reconstruction with esophagectomy for EC. To ensure a tension‐free cervical or intrathoracic anastomotic site and smooth passage of food in the gastric tube, Zhang et al reported that reconstructing the gastric tube as narrowly as possible will improve the outcome.^15^, ^16^

For over 10 years, we have used subtotal stomach (SS) reconstruction and hand‐sutured cervical esophagus‐subtotal gastric anastomosis at the neck wound in EC surgery. To the best of our knowledge, no previous reports have discussed the short‐ and long‐term outcomes, such as anastomotic leakage rate, QOL, and changes in body and muscle weight, of procedures using SS. The object of the present study was to assess the safety and feasibility of reconstruction using SS in EC surgery.

## 2 METHODS

### 2.1 Patients

All patients were staged preoperatively according to the 7th edition of the American Joint Committee on Cancer tumor‐node‐metastasis (TNM) classification.^17^ Clinical data were collected from a database of included patients at Gifu University Hospital.

### 2.2 Perioperative management

Before surgery, nutritional management, rehabilitation, and oral care were introduced from the first visit. A nasogastric feeding tube was inserted to the stomach in patients who could not take solid foods orally, and enteral feeding was administered for 24 hours.^18^ Further, to avoid oral mucosal damage during triple‐combination chemotherapy, we added an elemental diet.^19^, ^20^, ^21^ Just before surgery started, all patients received methylprednisolone (250 mg/body) intravenously. After surgery, the patient was transferred to the intensive care unit (ICU). Extubation was performed, and ambulation was started on postoperative day (POD) 1. A cannula was inserted into the cricothyroid ligament in all patients under local anesthesia, and regular endotracheal sputum suction was performed. The patient was transferred out of the ICU, and enteral feeding via jejunostomy tube was started on POD 2. Swallowing fluoroscopy using gastrografin was performed on POD 6 or 7, and food intake was started. Enteral nutrition via the jejunostomy tube was intermittently administered twice a day, and continued for 6 months after surgery. In principle, proton pump inhibitors were used for 6 months after surgery.

### 2.3 Transthoracic esophagectomy procedure

Treatment was in accordance with Japanese EC treatment guidelines.^22^ Esophagectomy was performed for advanced EC of clinical stages II or III after two courses of neoadjuvant chemotherapy. For patients with clinical nearly T4 tumor, surgery was performed when induction chemotherapy was effective and resection seemed possible.

In this study, the McKeown systemic approach was used for cases in which the upper mediastinal lesion's lymphadenectomy is required with the primary lesion located from upper thoracic to lower thoracic lesion. We performed a thoracic approach before the cervical and upper abdominal approaches. The basic operative procedure was a thoracoscopic subtotal esophagectomy with the patient in the left lower hemi‐prone position. Right thoracotomy was selected for clinical T3 cases, but even for these T3 or nearly T4 cases at the first visit, a thoracoscopic approach was selected if preoperative chemotherapy was mostly effective.

As reconstruction was basically performed along the posterior mediastinum route, the azygos vein arch and the right bronchial artery were dissected. Three‐field lymphadenectomy was performed when the main tumor was localized in the upper or middle thoracic esophagus and upper and cervical lymph nodes were swollen with the lower thoracic tumor. However, two‐field lymphadenectomy was performed when the main tumor was located in the lower esophagus without upper mediastinal and supraclavicular lymph node swelling, but additional neck lymph node dissection was performed if metastasis was revealed in the intraoperative frozen rapid histopathological results of lymph nodes around the recurrent laryngeal nerves. We routinely performed complete lymph node dissection around the recurrent laryngeal nerves from the upper mediastinum to the cervix as caudally as possible. The substernal route was selected for patients considered possible candidates for postoperative radiation. A jejunostomy was constructed in all cases. In patients undergoing reconstruction via the substernal route, a jejunostomy was inserted via the stomach.

### 2.4 Reconstruction technique using SS

The abdominal approach was performed via an upper abdominal wall incision with 9‐cm‐long axis. A laparoscopic hand‐assisted method was used in some patients with thick subcutaneous fat via a 7‐cm skin incision. Reconstruction was performed by two surgeons.

#### 2.4.1 Creation of SS

**Figure 1:**
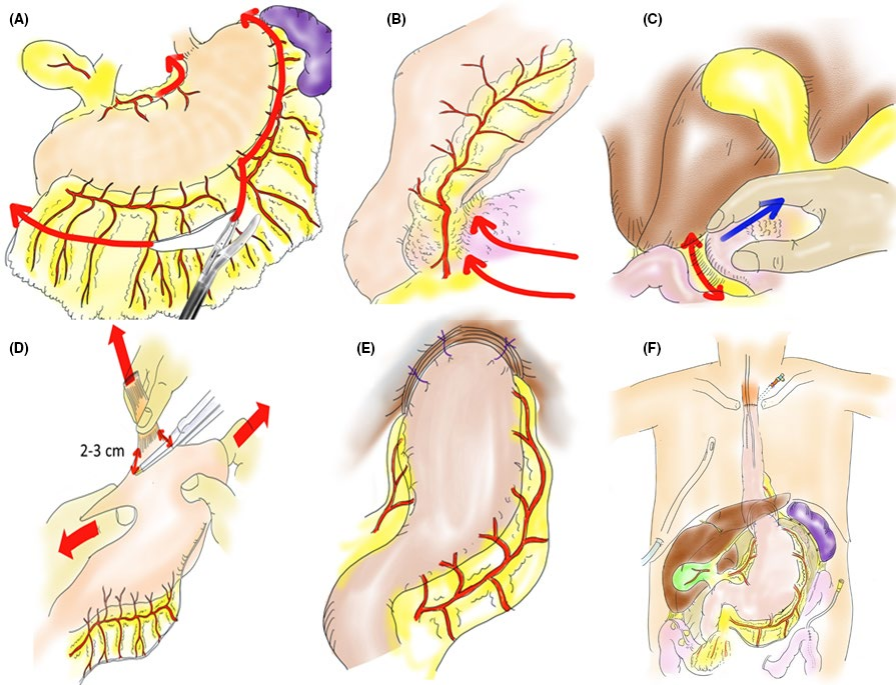
How to create a stomach tube. A, On the lesser curvature side, lymph node dissection including blood vessels of three branches from the afferent region of the left gastric artery is performed. Then, the greater omentum is dissected at a position about 5 cm distal to the right gastroepiploic artery and from the point of intersection of the left and right gastroepiploic arteries to the left side, and the entire greater omentum is then dissected along the gastric wall. B, Separate the pancreatic anterior fascia and physiological gastric adhesion sufficiently to increase elevation of the gastric tube. C, Kocher's mobilization is required to sufficiently elevate the stomach. D, Stapling: first, the operator fully extends his/her hands in the lengthwise direction, and then the assistant operator pulls the esophagus at right angles to that (this step must be followed). E, After creating the anastomosis, pull the stomach to the ventral side, especially the greater curvature side, and secure the stomach with three stitches in the crura of the diaphragm. F, Finally, a 12 french neraton catheter is placed as an information drain from the neck to the anastomotic site

The right gastric and right gastroepiploic arteriovenous arcades are preserved. The greater omentum is dissected 5 cm distal to the right gastroepiploic artery. The greater omentum is dissected along the gastric wall from the end points of the left and right gastroepiploic arteries to the top of the left gastric wall. The short gastric arteriovenous inflow site on the dorsal side of the stomach is also dissected just at the stomach wall (Figure 1A). The fusion of the mesocolon to the duodenum is dissected. We sufficiently separate the pancreatic anterior fascia due to its physiological adhesion to increase the range of motion at the back of the stomach (Figure 1B). We perform Kocher's mobilization to improve the elevation of the SS (Figure 1C). Lymph node dissection of the upper margin of the pancreas and ligation and dissection of the left gastric artery and coronary vein are then performed. The peritoneal incision is extended in the direction of the right diaphragm, and en bloc dissection of the lymph nodes is completed from in front of the aorta. Lymph node dissection on the left side of the upper pancreas is performed, leading to the cut surface of the left diaphragm. We dissect three branches from the afferent region of the left gastric artery to efferent direction for lesser curvature lymphadenectomy. The stomach is dissected with a stapler at a distance of about 2‐3 cm from the esophagogastric junction; actually, this is the difference from ‘Hole stomach’. When the advanced tumor located mainly in lower thoracic esophagus and slightly reached toward abdominal esophagus, lesser curvature side lymph node dissection is widely performed except for preserving only the first branch of the right gastric artery. In such cases, we resected the stomach with a distance of 5 cm from the lower end of the tumor to create a SS. In principle, SS reconstruction has not been performed for abdominal esophageal cancer. When the stomach is dissected, stapling is performed with both hands of the operator fully extending the stomach in the long‐axis direction (Figure 1D). The space of the crura of the diaphragm leg is the lateral width of three fingers. The SS is lifted to the neck by gently pushing from the abdomen, rather than pulling the SS strongly in the neck direction, while simultaneously not twisting it.

#### 2.4.2 Cervical esophagus‐subtotal stomach anastomosis

**Figure 2:**
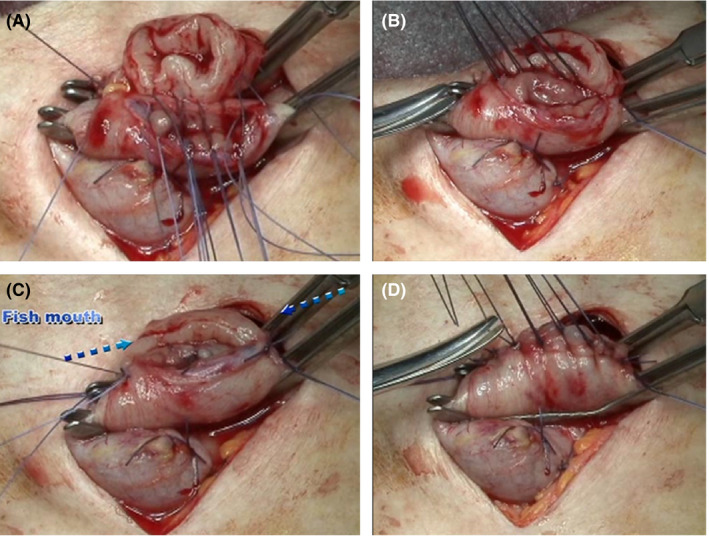
Anastomosis of the cervical portion. A, Posterior suture: perform interrupted suturing of the esophageal outer membrane, muscle layer and gastric seromuscular layer from the left to right side using about 10 needles. B, Lift and pull only the left and right ends of sutures and cut the remaining sutures. C, Perform running suture of the esophageal membrane and gastric membrane from the center to left side and ligate with suture thread that is inserted from the outer to inner and inner to outer side at the edge of the left side. Next, perform running suture from the center to the right side and suture the edge similarly. At this point, both ends look like a fish mouth. D, Anterior suture: perform interrupted suture with modified Gambee's anterior suture using about 7‐8 needles

In the case of two‐field dissection, make an oblique incision in the left neck. Use satinsky vascular forceps (04‐31‐M; Tanaka Medical Instrument Co., Ltd.) to grasp the cervical esophagus and the SS. From the left to the right side of the posterior wall, ligate and suture the seromuscular layer of the SS and the adventitia muscle layer of the cervical esophagus with 3‐0 absorbent suture (Figure 2A). As a guide, the left and right edge pitch should be 3 mm, and other pitches should be 4 mm (Figure 2B). A continuous suture of the mucosa and submucosa is performed from the center of the rear wall to the left side. At the left end, full‐layer sutures are added in the order of outer and inner and outer, and are ligated with outer continuous suture. Perform the same procedure on the right side of the rear wall. At the end of the posterior wall anastomosis, the anastomosis looks like a fish mouth (Figure 2C). The anterior wall is sutured from left to right with a modified Gambee method (Figure 2D). Then pull the stomach to the ventral side, and secure the SS with three stitches in the crus of the diaphragm to prevent postoperative gastric invasion into the thoracic cavity and excessive dilation in the mediastinum. Take extreme care at this time to pull down the greater curvature side of the SS, which tends to be located on the back side by weight. This results in the SS being linearized in the thoracic cavity (Figure 1E). Finally, a size 12 French neraton catheter is placed as an information drain from the neck to the anastomotic site (Figure 1F).

### 2.5 Postoperative complications and quality of life measurement

Patients without leakage shadow at gastrografin test POD 6 or 7 or treatment for leakage including conservative treatment were defined as no anastomotic leakage. Other postoperative complications were categorized using the Clavien‐Dindo classification.^23^ Patient QOL was measured using the esophageal site‐specific module of the validated European Organization for Research and Treatment of Cancer Quality of Life Questionnaire (EORTC QLQ‐OES18)^24^ at postoperative months 3 and 6, and years 1, 3, and 5. Esophagitis or residual esophageal and gastric cancer were checked routinely by endoscopy every year for 5 years after surgery.

Body and skeletal muscle weights were measured using an In Body 720^®^ (FUJITEX Corporation) at pretreatment, after NAC, and at 3 and 6 months and years 1, 2, 3, 4, and 5. QOL surveys and body weight and skeletal muscle mass were surveyed in all patients at prescribed points in 300 postoperative patients. In this study, we aggregated only 5‐years survivors, excluding recurrent cases, to observe the QOL and body weight changes using this operative procedure purely.

### 2.6 Statistical analysis

Results are expressed as the median (interquartile range) for quantitative variables and percentage for qualitative variables. The *χ* ^2^ test or Fisher exact test was used for categorical variables, and the nonparametric Wilcoxon rank sum test was used for continuous variables. A *P*‐value < .05 was considered significant. All statistical analyses were performed with the SPSS 20.0 software package (SPSS).

### 2.7 Ethical considerations

This study was conducted in accordance with the World Medical Association Declaration of Helsinki. This study was approved by the review board of the Gifu University Hospital (Approval no. 2019‐170).

## 3 RESULTS

### 3.1 Patient characteristics

Between January 2008 and September 2019, 320 patients with EC underwent thoracic esophagectomy at Gifu University Hospital. Seventeen patients were excluded because they underwent reconstruction using ileocolic intestine or jejunum, as were three patients who underwent jejunal reconstruction. Consequently, 300 patients with EC who underwent reconstruction using SS were enrolled in the present study. Patient demographic and clinical characteristics are shown in Table 1. As for histological tumor type, 295 cases (98.3%) were squamous cell carcinoma and five cases (1.67%) were adenocarcinoma in which main tumors were located in the lower thoracic esophagus.

**Table 1:** Patient Characteristics and Postoperative Complications (n = 300)

Characteristic	
Age^a^ (y)	67.6 ± 9.1
Sex (Male/Female)	252 (84%)/48 (16%)
Performance Status (0/1/2/3)	32 (10.7%)/246 (82.0%)/21 (7.0%)/1 (0.3%)
Tumor location (Ut/Mt/Lt)	43 (14.3%)/171 (57.0%)/86 (28.7％)
Clinical stage^b^ (I/II/III/IV)	72 (24%)/42 (14%)/155 (51.7%)/31 (10.3%)
Histological tumor type (SCC/AC)	295 (98.3%)/5 (1.67%)
Preoperative therapy (CT/CRT/None)	189 (63%)/30 (10%)/81 (27%)
Thoracic approach (Right thoracotomy/Thoracoscopic)	210 (70%)/90 (30%)
Lymph node dissection (Two fields/Three fields)	85 (28.3%)/215 (71.7%)
Route of reconstruction (Substernal/Posterior mediastinal)	49 (16.3%)/251 (83.7%)
Operative time (minutes)^a^	378 ± 92.0
Blood loss (g)^a^	183 ± 99.6

Postoperative complications	No. (%)
Early‐phase complications	
Anastomotic leakage	2 (0.67)
Vocal cord palsy	33 (12.0)
Atrial fibrillation	21 (7.0)
Delirium	10 (3.3)
Pneumonia	9 (3.0)
Chylothorax	9 (3.0)
Pulmonary thromboembolism	3 (1.0)
Ileus	3 (1.0)
Postoperative bleeding	2 (0.67)
Hepatic portal venous gas	1 (0.33)
Sick sinus syndrome	1 (0.33)
Esophageal hiatal hernia	1 (0.33)
Air leakage	1 (0.33)
Reoperation: air leakage, chylothorax, esophageal hiatal hernia	3 (1.0)
Hospital mortality	0 (0.0)
Late‐phase complications	
Stenosis of anastomotic site	15 (5.0)
Nausea	11 (3.7)
Dumping syndrome	7 (2.3)
Ulceration of subtotal gastric tube	1 (0.33)

Abbreviations: Ut, Upper esophagus; Mt, Middle esophagus; Lt, Lower esophagus; SCC, Squamous cell carcinoma; AC, adenocarcinoma; CT, Chemotherapy; CRT, Chemoradiotherapy.

^a^ Mean ± standard deviation.

^b^ TNM Classification of Malignant Tumors, 7th ed.

### 3.2 Surgical characteristics

Surgical characteristics are shown in Table 1. The posterior mediastinum route was used in 251 (83.7%) patients, and the substernal route was used in 49 (16.3%) patients. Three‐field lymphadenectomy was performed in 215 (71.7%) patients, and two‐field lymphadenectomy was performed in 85 (28.3%) patients. Ninety (30%) patients underwent subtotal esophagectomy via thoracoscopy.

### 3.3 Postoperative complications

Anastomotic leakage was observed in two patients (0.67%). Other postoperative complications classified as Grade II or more are listed in Table 1. Pneumonia was observed in nine patients (3.0%). Fifteen patients (5.0%) had an anastomotic stenosis requiring a bougie. Nausea occurred in 11 patients (3.7%), and dumping syndrome occurred in seven patients (2.3%). There was no operative mortality.

### 3.4 Evaluation of QOL, body weight, and muscle weight

**Figure 3:**
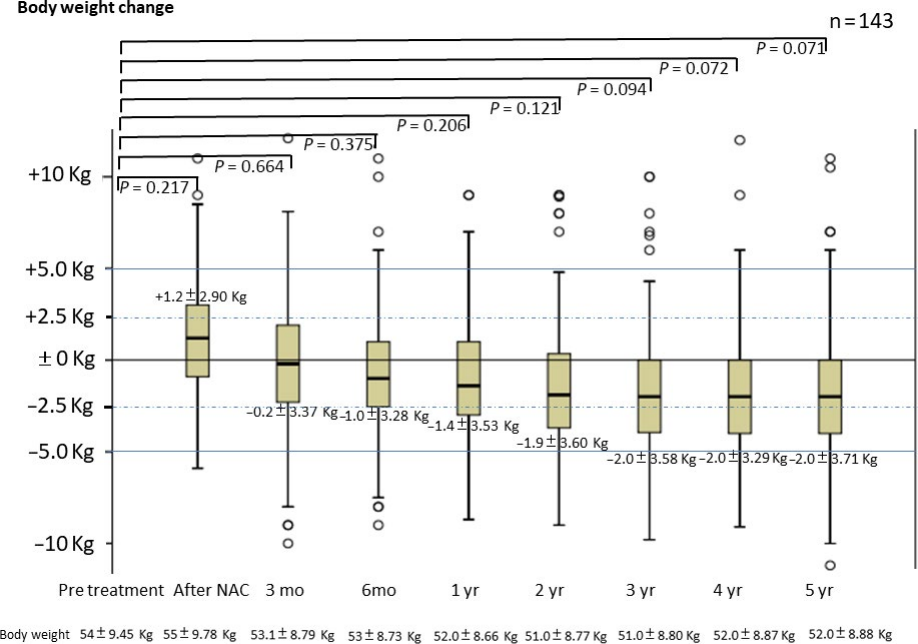
Change in body weight after surgery

**Figure 4:**
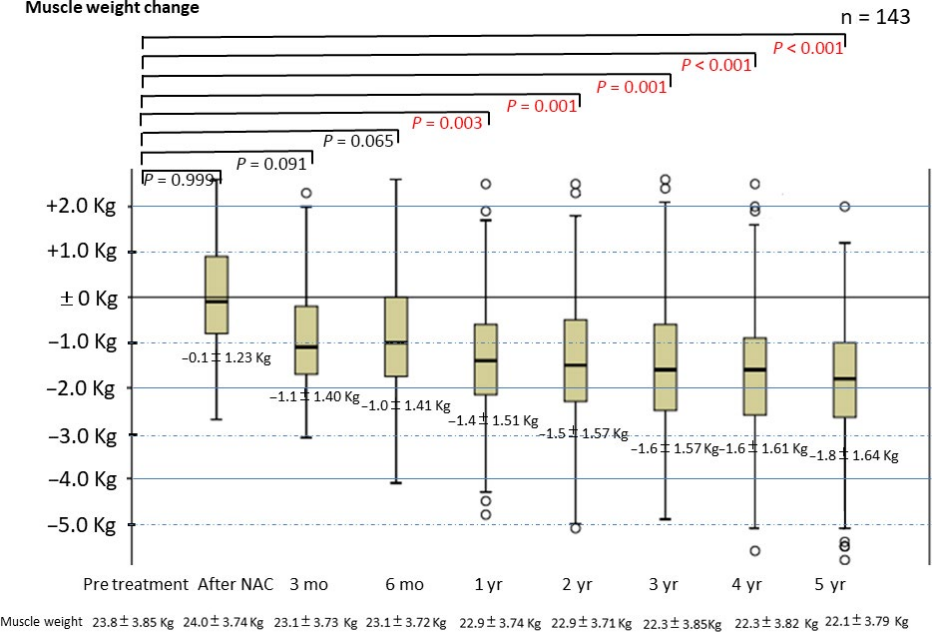
Change in muscle weight after surgery

We interviewed and measured 143 patients (123 [86.9%] men and 20 [14.0%] women) who survived for more than 5 years at the time of this report. Cancer stages of the 143 patients were: I, 55 (38.5%); II, 32 (22.4%); III, 52 (36.4%); and IV, 4 (2.8%) patients. Respective EORTC QLQ‐OES18 scores (average ± standard deviation) of these 143 patients at postoperative months 3 and 6 and years 1, 3, and 5 were as follows: dysphagia score: 1.82 ± 0.40, 1.78 ± 0.42, 1.15 ± 0.42, 1.15 ± 0.44, and 1.0 ± 0.1; early feeling of abdominal fullness score: 2.03 ± 0.65, 1.89 ± 0.46, 1.30 ± 0.39, 1.22 ± 0.41, and 1.09 ± 0.38; feeling of reflux score: 1.29 ± 0.37, 1.20 ± 0.32, 1.10 ± 0.11, 1.10 ± 0.09, and 1.09 ± 0.07. Proton pump inhibitors were used for 6.2 ± 2.2 months after surgery. In the Los Angeles classification,^25^ the incidence of reflux esophagitis occurred with Grade 0/A/B/C/D in 131 (91.6%)/10 (7.0%)/2 (1.4%)/0/0 in the first year. Subsequent observations did not show Grade C or D. Grade A was two (1.4%) in the second year, one (0.7%) in the third year, two (1.4%) in the fourth and fifth years. Grade B was two (1.4%) in the second year, three (2.1%) in the third year, one (0.7%) in the fourth and fifth years, and Grade 0 in the rest. Figures 3 and 4 show the changes in the 143 patients’ body and skeletal muscle weights, respectively. The patients’ body weights were as follows: pretreatment, 54 ± 9.45 kg; postoperative year 1, 52 ± 8.66 kg; postoperative year 3, 51 ± 8.80 kg; and postoperative year 5, 52 ± 8.88 kg. Body weight decreased an average of −2 ± 3.71 kg (*P* = .071) over the 5 years, and skeletal muscle weight decreased significantly after 1 year (*P* = .03).

## 4 DISCUSSION

There are various reconstruction methods for EC surgery,^26^ and many methods have been investigated in terms of blood flow in the stomach.^27^ To our knowledge, this is the first report to investigate the short‐ and long‐term outcomes of patients undergoing the procedure of SS. Five factors affect safety and good QOL: (a) the blood vessel network in the stomach; (b) methods to increase the distance to the anastomotic site at the neck,; (c) hand‐sewn anastomosis that looks like a fish mouth; (d) straightening of the SS in the thoracic cavity; and (e) locating the anastomotic site just around the circumference of the sternum and vertebral body.

Narrow gastric tube reconstruction and stapled anastomosis are mainly performed in many countries. By using the junction between the left gastroepiploic and short gastric vessels via the splenic hilar vascular arcade, the distal portion of the gastric tube can be sufficiently nourished. Double‐stapling, purse‐string, side‐to‐side linear‐stapled, and end‐to‐side hand‐sewn methods of anastomosis are reported for EC.^28^

**Figure 5:**
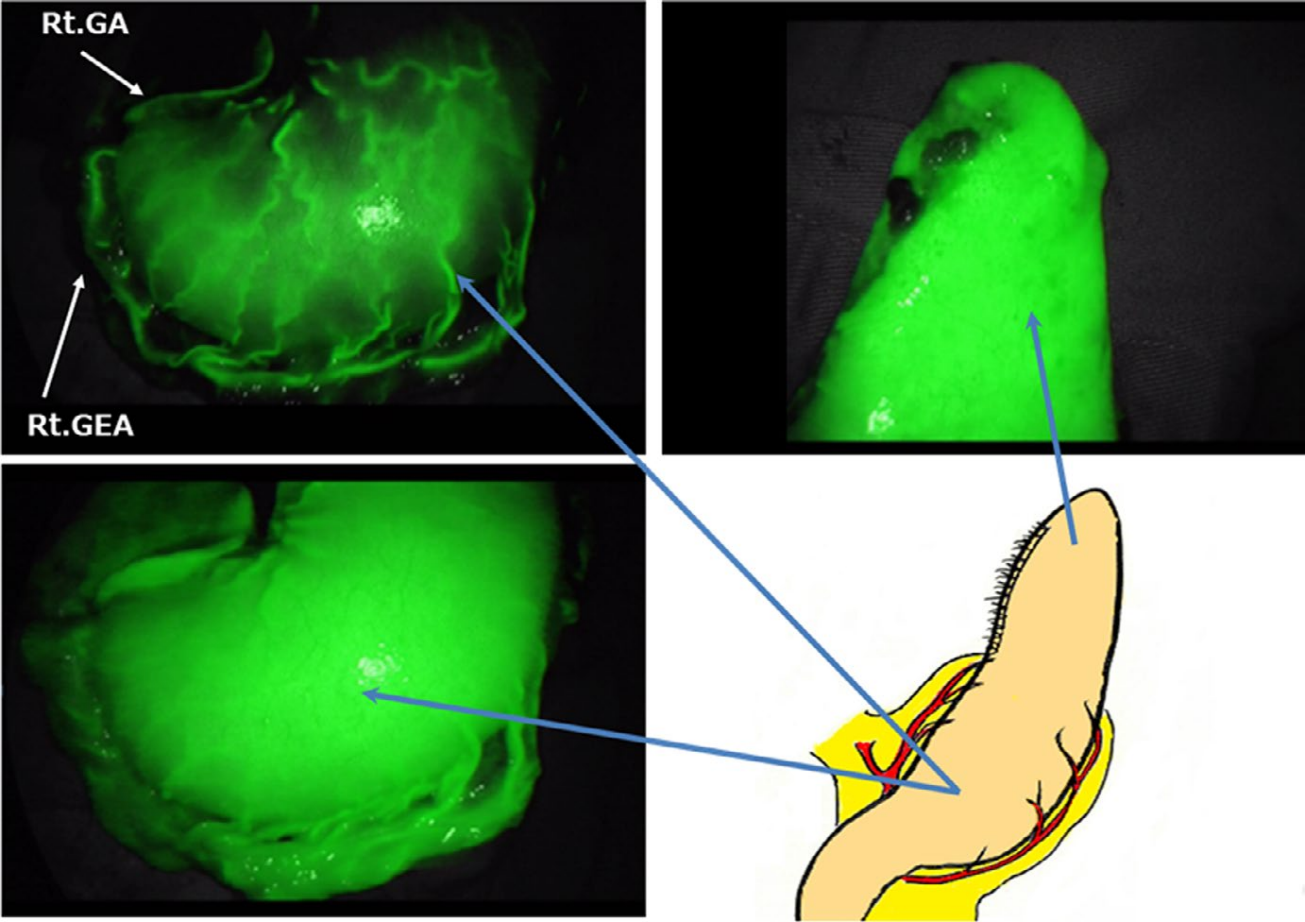
Circulatory network in the subtotal stomach as revealed by indocyanine green (ICG) fluorescence method. Rt. GA: right gastric artery, Rt. GEA: right gastro‐epiploic artery

The greatest benefit of reconstruction using SS is good blood flow. Although it appears advantageous that the length of the reconstructed stomach can be extended, the blood flow at the tip of the narrow gastric tube may not be sufficient. We assume that one cause is blockage of the abundant vascular network in the submucosal layer of the entire stomach when creating a narrow gastric tube. The stomach has excellent blood flow at the anastomotic site due to preservation of the network of blood vessels in the stomach wall.^29^ Thus, blood flow to the tip is sufficiently maintained by using the right gastric and right gastroepiploic arteriovenous arcades as the blood supply and drainage source. Although we did not use indocyanine green (ICG) in 299 cases, we experimentally performed intraoperative bloodstream evaluation of SS using ICG fluorescent imaging in one case (Figure 5). The images show a vascular network under the gastric mucosa and good blood flow up to the tip of the SS. Thus, we believe that good blood flow in the SS is the biggest factor in our minimal suture failure rate of <1%.

Additionally, the difference between an intrathoracic anastomosis and our technique is that the anastomotic site is located just around the circumference of the sternum and vertebral body, which may reduce leakage.

**Figure 6:**
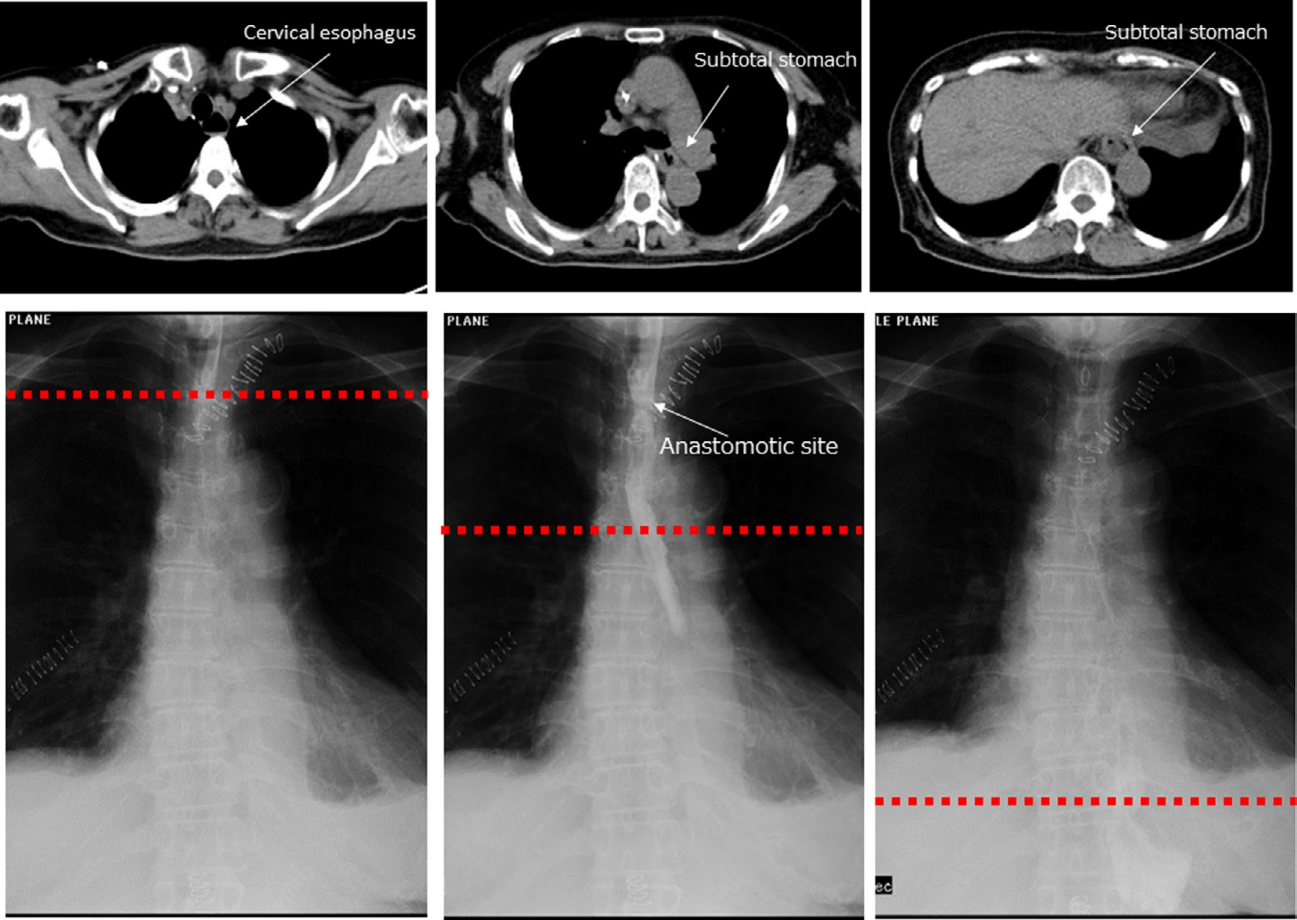
Swallowing test using gastrografin after reconstruction along with contrast‐enhanced computed tomography. Red dotted line indicates the height of the computed tomography slice. The reconstructed stomach is seen located in the original esophageal position without bulging, and gastrografin flows straight down

However, there are two disadvantages of reconstruction using SS. The first is the tension created when raising the SS to the anastomotic site at the neck because the SS has a shorter reconstructed organ length compared with that of the narrow gastric tube. However, by removing physiological adhesions on the posterior side of the stomach wall and using Kocher's mobilization, we can raise the SS sufficiently to the neck. The second is deflection or deformation of the SS in the thoracic cavity. Expansion of the SS in the thoracic cavity can result in delayed emptying of the stomach contents, which may occur because the SS is not pulled sufficiently toward the abdominal side after the cervical anastomosis. Therefore, after anastomosis, we pull the SS toward the abdominal side, especially the greater curvature side of the stomach, and straighten it, and then suture the SS to the crura of the diaphragm. This prevents both excessive expansion of the SS in the thoracic cavity and intrusion of the reconstructed stomach into the thoracic cavity. We pull the greater curvature side, which is located dorsally in the thoracic cavity due to its weight, with great care because the determinant of the distance of the SS is defined by the length of the smaller curvature side. If these two procedures are followed, gastrografin on postoperative oral fluoroscopy flows straight through the thoracic cavity at the original thickness of the esophagus as if the SS is occupying the original esophageal position (Figure 6).

Finally, and this is not described in any previous report, when using SS for esophageal surgery, the range of motion of the right hand of the operator who pushes up a large SS toward the anastomotic site is limited unless a minimum 7‐cm abdominal open wound is created. Therefore, it is not recommended to lift up the SS through the thoracic cavity to the neck under complete laparoscopic surgery. Of course, with an abdominal incision of at least 7‐9 cm, the abdominal lymph nodes can be dissected under direct vision.

Two patients suffered anastomotic leakage in the present study. One patient developed EC after surgery for rectal cancer and had poor nutritional status according to continuing adjuvant chemotherapy for 1 year. The second patient suffered severe pneumonia on the first postoperative day and was treated with methylprednisolone steroid pulse therapy at 1000 mg for 3 days.

There are some methods of hand sewing and instruments used in anastomosis that have both advantages and disadvantages. The reasons for our selection of hand‐sewn method with layer‐to‐layer anastomosis are as follows: emphasis on the neovascular network; end‐to‐end anastomosis eliminates the ischemic area at the anastomotic site; inverting anastomosis by forming a fish mouth by hand‐suture that is the basis of intestinal anastomosis; the direct view of the cervical anastomosis site for both the operator and assistant will be instructive for the young surgeons; and it is easy to perform Bougie for anastomotic stenosis, because it does not use staples. If the leakage rate would be <1% like this report, our report, mechanical anastomosis can be considered for SS too, but still hand sewing will be better because SS has a margin of lifting about 2.5‐3.0 cm in the cervical portion for anastomosis. At that distance it will be difficult to insert the instrument.

Regarding postoperative morbidity, narrow gastric tube reconstruction is considered to cause fewer postoperative lung disorders compared to SS reconstruction.^11^ However, pneumonia occurred postoperatively in only nine patients (3.0%) in the present study, which may indicate that our technique properly prevents SS deflection in the thoracic cavity. Proper postoperative oral care and speech and physiological therapy also help to prevent postoperative lung complications.

Narrow gastric tube reconstruction is reported to result in less postoperative reflux and better QOL than whole gastric tube reconstruction.^15^Dysphagia and early feeling of abdominal fullness scores tended to be high after surgery but gradually decreased after 6 months. Good results were obtained for postoperative symptoms of reflux as assessed by the QOL test. If we follow these procedures, we will maintain the benefits of subtotal gastric blood flow preservation, low rate of complaints of duodenogastroesophageal reflux and slow emptying of the intrathoracic stomach. From the QOL questionnaire results, we could see that complaints about those issues were mild and actual endoscopic findings showed low rate of reflux esophagitis. The most important difference from the previous reports of SS is that our SS has a width like a narrow gastric tube in the thoracic cavity (Figure 6) and the stomach body is located in the abdominal cavity after anastomosis (Figure 1F). Proton‐pump‐inhibitors were administered for about half a year. The reflex score tends to decrease even after stopping the drug, and further administration may not be required.

The factors for maintaining postoperative weight in patients with EC are thought to be the shape of the reconstructed SS and the continuation of oral nutritional supplements from the preoperative to postoperative period. Although the maintenance of postoperative QOL is greatly influenced by perioperative management including nutritional management and speech and physiological therapy, we believe that SS reconstruction created according to our method is the basis for this.

The body weight of the survivors was maintained for 5 years after esophagectomy although skeletal muscle weight was significantly reduced at 1 year after surgery. Given that the skeletal muscles of the elderly decrease by 3% over 2 years around the age of 70, this does not seem to be a problem.^30^ However, we have started individual nutritional management and physical therapy using a social network service. Patients participate in a network, a completely private social network service called 'Medical Care Station' (Embrace Co., Ltd.), of attending physicians, pharmacists, supervising dietitians, and rehabilitation technicians. Under strong security, all medical staff will support the trivial patient's dietary, nutritional, and rehabilitative questions postoperatively through their mobile phones. In response to the patient's complaints, we provide dietary guidance and reintroduction of physical therapy. Postoperative complaints in most cases are resolved by 1 year after surgery; however, it is important to deal individually with the rare patient with a poor understanding of how to eat and the importance of exercise. ^31^

A jejunostomy tube was inserted into jejunum in 251 cases. In one case, the operation was performed by wrapping and twisting the intestinal tract at the insertion site. No other troubles, including infectious ones, occurred. The following points should be noted in our department when inserting a jejunostomy tube. The tube is inserted at 40 cm anal side from the Treitz ligament, and a 20 cm length is inserted into the jejunum. If it is longer than 40 cm, the oral side of jejunum may be twisted. The tube penetrates the abdominal wall diagonally on the caudal side of the Treitz ligament. This is to prevent the small intestine from becoming Z‐shaped at the inserted site. It is fixed to the abdominal wall after suturing over 10cm by the Witzel method.

In oncological aspect, the local recurrence at the anastomotic site is zero in 300 cases. In terms of the resected distance of the oral stomach for the SS, it is unlikely that the SS would be at risk of recurrence as a reconstructed stomach. In addition, no recurrence of the lymph nodes in the lesser curvature side of the stomach occurred, which may be due to sufficient lymph node dissection of the lesser curvature side.

This study has several limitations. First, this is a single‐institution study. Second, other techniques were not evaluated prospectively. However, among these 300 cases of EC surgery consecutively performed in our institution in which SS was intended to be used, no conversion to another technique was made because of problems during surgery and there was no change about operative methods, devices, and perioperative managements.

## 5 CONCLUSION

We believe that reconstruction using SS is not only safe but also contributes to the maintenance of long‐term QOL and body weight of patients receiving EC surgery. Future randomized trials of SS and narrow gastric tube reconstruction are desired.

## DISCLOSURES

Funding: Dr Yoshida reports receipt of grants, personal fees and non‐financial support from EA Pharma Co., Ltd., Sanofi, Yakult Honsha Co., Ltd., Chugai Pharmaceutical Co., Ltd., Taiho Pharmaceutical Co., Ltd., Takeda Pharmaceutical Co., Ltd., Eli Lilly Japan KK, Daiichi Sankyo Co., Ltd., Ono Pharmaceutical Co., Ltd., Merck Serono Co., Ltd., and Novartis Pharma KK; and grants from Kyowa Hakko Kirin Co., Ltd. These funding sources had no role in the design, practice, or analysis of this study.

Conflict of Interest: All of the other authors declare that they have no conflicts of interest.
